# Dissecting the null model for biological invasions: A meta-analysis of the propagule pressure effect

**DOI:** 10.1371/journal.pbio.2005987

**Published:** 2018-04-23

**Authors:** Phillip Cassey, Steven Delean, Julie L. Lockwood, Jason S. Sadowski, Tim M. Blackburn

**Affiliations:** 1 School of Biological Sciences and the Environment Institute, The University of Adelaide, Adelaide, Australia; 2 Department of Ecology, Evolution and Natural Resources, Rutgers University, New Brunswick, New Jersey, United States of America; 3 Bodega Marine Lab, University of California at Davis, Bodega Bay, California, United States of America; 4 Department of Environmental Science and Policy, University of California at Davis, Davis, California, United States of America; 5 Department of Genetics, Evolution & Environment, Centre for Biodiversity & Environment Research, University College London, London, United Kingdom; 6 Institute of Zoology, Zoological Society of London, Regent’s Park, London, United Kingdom; Estación Biológica de Doñana (EBD-CSIC), Spain

## Abstract

A consistent determinant of the establishment success of alien species appears to be the number of individuals that are introduced to found a population (propagule pressure), yet variation in the form of this relationship has been largely unexplored. Here, we present the first quantitative systematic review of this form, using Bayesian meta-analytical methods. The relationship between propagule pressure and establishment success has been evaluated for a broad range of taxa and life histories, including invertebrates, herbaceous plants and long-lived trees, and terrestrial and aquatic vertebrates. We found a positive mean effect of propagule pressure on establishment success to be a feature of every hypothesis we tested. However, establishment success most critically depended on propagule pressures in the range of 10–100 individuals. Heterogeneity in effect size was associated primarily with different analytical approaches, with some evidence of larger effect sizes in animal rather than plant introductions. Conversely, no variation was accounted for in any analysis by the scale of study (field to global) or methodology (observational, experimental, or proxy) used. Our analyses reveal remarkable consistency in the form of the relationship between propagule pressure and alien population establishment success.

## Author summary

Alien species are a major contributor to human-induced global environmental change. The probability of whether or not an alien species will successfully establish in a novel environment is often related to the number of times a species is introduced and the number of individuals that are introduced each time, collectively termed ‘propagule pressure’. Despite this evidence, we don’t yet know whether this is a universal characteristic of species invasions, and the role of propagule pressure continues to be questioned. Here, we present a quantitative meta-analysis of the relationship between propagule pressure and establishment success across a broad range of species and geographies. We found that propagule pressure was consistently and positively associated with the establishment success of alien species. We conclude that propagule pressure is indeed the most consistent and strongest determinant of alien species establishment. No other factors suggested to explain establishment success can claim such universal support. Our results underpin a clear policy and management target for slowing invasion rates by reducing propagule pressure—ideally to single figures or zero—regardless of any other feature of the invasion.

## Introduction

Alien species constitute a major threat to global biodiversity [1–4], and some impose substantial socioeconomic and management costs [5–8]. Yet not all species transported beyond their native ranges establish viable alien populations, and this variability has motivated a sustained search for factors that distinguish those successful alien populations from those that fail [9, 10]. One factor that has received considerable scrutiny is the total number of individuals that are introduced to found an alien population, termed propagule pressure [11]. While extinction is the ultimate fate of all populations and species, the basic principles of conservation biology attest that, given suitable environmental conditions (and all else being equal), the probability of a natural population persisting over some period of time is a positive function of population size [12]. If alien populations are beholden to the same rules, then we expect their establishment success in suitable environments to be a positive function of propagule pressure. A growing body of studies has explored this relationship, and positive effects have been reported consistently enough that the influence of propagule pressure has been argued to be a ‘null model for biological invasions’ [13].

Despite all the attention paid to the effect of propagule pressure on alien population establishment success, there are still substantial and surprising gaps in our knowledge of this relationship. Most notably, there has been no quantitative assessment of evidence for the strength, shape, or variation in the statistical effect of propagule pressure. Given that propagule pressure underpins our basic understanding of the ecological and evolutionary dynamics of small founding populations [14], as well as informing the management of intentional biocontrol introductions [15, 16] and influencing import risk assessments and biosecurity planning [17, 18], this gap represents a glaring omission from the literature. Here, we provide the first quantitative meta-analysis of the growing evidence base on propagule pressure, which addresses these gaps and identifies opportunities for deeper exploration of the propagule pressure effect and its application.

### Sources of variation in propagule pressure

Increases in propagule pressure can be achieved through the release of large numbers of individuals (i) in any single introduction event (propagule size), (ii) through many independent introduction events (propagule number), or (iii) through a combination of the two (propagule pressure) [11]. Positive effects on establishment success have been found for all three components (e.g., see Table 3.1 in [19]), in which establishment is defined as an alien population that is self-sustaining but not necessarily spreading. In addition, some studies have suggested that there is a relatively narrow critical range of propagule sizes over which variation is especially important for establishment success [20]. Yet few studies have quantified, and none have quantitatively compared, effect sizes for relationships between establishment success and propagule number, size, and pressure [21].

Reported relationships between propagule pressure and establishment success range from strongly positive [22] through indistinguishable from zero [23], to even negative [24]. However, to date, no one has identified the possible sources of this variation nor quantified how (or if) a source could influence reported relationships. The relationship between propagule pressure and establishment success has been evaluated for a broad range of taxa and life histories, including small aquatic invertebrates [25], terrestrial herbaceous plants [26], long-lived trees [27], and terrestrial and aquatic vertebrates [28, 29]. There is sound evidence that species-level and location-level factors can influence establishment success [30]. Thus, given the vast array of life histories and tremendous variety of ecological contexts contained within this suite of studies, we predict that the propagule pressure effect will vary in magnitude and perhaps direction (i.e., positive or negative) between them. In addition, the effect has been studied across an extraordinary range of spatial scales spanning experimental field plots and mesocosms [31] up to worldwide surveys [11, 14, 32]. The propagule pressure effect represents a population-level process; for this reason, we predict that studies conducted at spatial scales on which data can be directly measured (e.g., using experimental approaches) will produce larger effect sizes. In contrast, global surveys and those that are observational undoubtedly rely on data that contain inconsistencies and reporting errors (e.g., [33]); these may serve to obscure underlying relationships between establishment and propagule pressure [34], likely lowering the overall strength of effect size estimates.

This variety of study designs, taxonomic diversity, and spatial scales has required variation in the statistical methodologies adopted. These methods range from simple univariate models to complicated multivariate structures that seek to disentangle the often complex intercorrelations between propagule pressure, species traits, and environmental conditions [35–38]. Although this variability in study designs and approaches has been long recognised [11, 14], there are no quantitative analyses that determine the influence these factors have on the strength or shape of the propagule pressure effect on the establishment success of alien species. In particular, we predict that analyses that isolate the effect of propagule pressure can produce larger effect sizes, due to omitted variable bias, than those that simultaneously evaluate other predictors.

Propagule pressure can be difficult to measure in the natural experiments that represent real-world introductions of alien species and impossible to estimate retrospectively. To deal with this challenge, proxies for components of propagule pressure are analysed instead of direct estimates of propagule pressure itself. Proxies are variables that are not measures of the number of individuals released to found different populations but that are thought to correlate with that number [39]. For this reason, we expect that results using proxy measures will produce relatively weaker effect sizes than those using direct measures, from either observational or experimental studies.

The observed variability in the strength of the relationship between propagule pressure and establishment success has led some authors to suggest that the proposed ‘null model for biological invasions’ [13] is actually an artefact of a prominent set of purportedly pseudoreplicated studies from a limited set of locations [40]. The prime example of this effect is the set of studies based on Acclimatisation Society introductions of alien vertebrates (particularly birds and mammals) to New Zealand [41, 42]. Analyses based on this dataset have revealed consistent support for the propagule pressure effect [43–48]. However, continued analysis of these data has led to the suggestion of a ‘Kiwi’ effect, in which the influence of propagule pressure has been substantially larger in New Zealand, particularly for Acclimatisation Society introductions of alien birds [48]. Although relationships between propagule pressure and establishment success have been reported for a range of locations, spatial scales, and data sources [14], the anecdotal evidence is insufficient to definitively state that the effect is generalisable in the absence of formal analysis of the heterogeneity among effect sizes and of the variation in these relationships. Our analyses directly test for generality in the propagule pressure effect.

### Meta-analysis of propagule pressure

Here, we have performed the first systematic and quantitative review of the relationship between propagule pressure and establishment success for alien species introductions. We used quantitative meta-analytical methods to estimate the overall effect of propagule pressure on establishment success and to test for heterogeneity in this relationship. We then assessed the extent to which heterogeneity can be explained by the set of a priori hypotheses concerning factors that vary across studies. Specifically, we tested whether the relationship between propagule pressure and establishment success differs among (i) taxonomic groups, (ii) studies performed at different spatial scales, and (iii) relationships analysed using proxies for propagule pressure compared with those studies using direct measures (i.e., observational and experimental studies, which themselves are expected to differ [49]). We also tested whether variation in the effect can be ascribed to features of the statistical analyses employed, specifically through either (iv) the number of predictors in the model (i.e., univariate versus multivariate analyses) or (v) the transform used to model propagule pressure (i.e., linear or log transformed). For the subsets of relevant studies, we assessed the strength of the relationship in studies of (vi) propagule number versus propagule size and (vii) different ranges of propagule size. Finally, for the small number of experimental studies (*n* = 11) that provided raw data on the binary outcome of establishment success (0 = failed; 1 = success), we directly evaluated the specific variation in the shape (i.e., slope) of the effect of propagule pressure on establishment probability.

## Results

**Fig 1:**
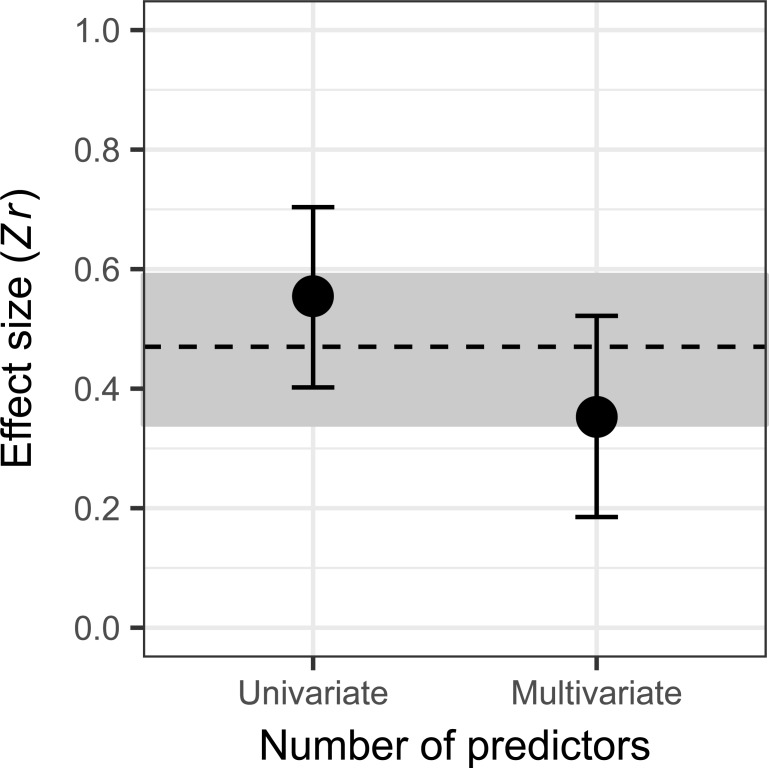
Differences in the effect size (*Zr*) for levels of the most influential moderator variable (‘Number of predictors’) in explaining heterogeneity in the complete dataset (*n* = 96; Δ_LOO-IC_ relative to Intercept-only model = 3.4; Table A in S1 Text). The dashed line shows the mean population-level effect size (0.47), and the grey bands show the 95% CI (0.34–0.59). CI, credible interval; LOO-IC, leave-one-out cross-validation information criterion; *Zr*, Fisher’s z-transformed correlation coefficient.

The overall population-level effect size (mean *Zr*) across all 56 studies was positive (0.47), with 95% Credible Intervals (CI) that did not overlap zero (95% CI = 0.34–0.59). However, these studies showed substantial heterogeneity among effect sizes (I^2^ statistics; Table 1; Fig B in S1 Text). Variation among effect sizes was primarily explained (Δ leave-one-out cross-validation information criterion [LOO-IC] > 2.0 from the Intercept-only model) by the larger estimates from univariate analyses compared with multivariate analyses (Fig 1) and secondarily by differences among taxa (Table A in S1 Text). Studies of plants produced smaller effect sizes than those of either vertebrate or invertebrate studies. No further moderator variables explained additional heterogeneity among effect sizes in the full dataset (Table A in S1 Text). There was no evidence of publication bias in the full dataset. The intercept from the modified Egger's regression test of residual effect sizes against their inverse precision was not significantly different from zero (Intercept = 0.36, *P* = 0.61; Fig C in S1 Text).

**Table 1:** I^2^ statistics and mean effect sizes for the full dataset and resulting subset analyses.

	Sample size	Mean effect size (*Zr*)	Among studies	Within studies	Residual error
		(*95% CI*)	Standard deviation [*I*^*2*^ *% heterogeneity*]		
Complete dataset (Table A in S1 Text)	96	0.47	0.20	0.41	0.08
		*(0*.*34*, *0*.*59)*	*[19*.*0]*	*[78*.*1]*	*[2*.*9]*
‘Number of predictors’			0.18	0.41	0.08
			*[16*.*2]*	*[80*.*8]*	*[3*.*0]*
Metric model (Table B in S1 Text)	70	0.48	0.26	0.38	0.10
		*(0*.*31*, *0*.*63)*	*[29*.*8]*	*[66*.*1]*	*[4*.*2]*
Propagule size model (Table C in S1 Text)	56	0.51	0.25	0.42	0.11
		*(0*.*33*, *0*.*68)*	*[24*.*7]*	*[70*.*8]*	*[4*.*5]*
‘Transform’ + ‘Propagule size’			0.29	0.36	0.11
			*[37*.*0]*	*[58*.*0]*	*[5*.*0]*

I^2^ statistics were calculated as the percent variation attributed to each component of variance (shown as standard deviations). For each analysis, the effect size and I^2^ statistics from the Intercept-only model, the moderators from the highest ranked model (if not the Intercept-only model), and the I^2^ statistics from the highest ranked model (with moderators) are shown. LOO-IC model-selection tables are provided in the supporting information (see Tables A–C in S1 Text).

**Abbreviations:** CI, credible interval; I^2^, heterogeneity; LOO-IC, Leave-one-out cross-validation information criterion; *Zr*, Fisher’s z-transformed correlation coefficient.

**Fig 2:**
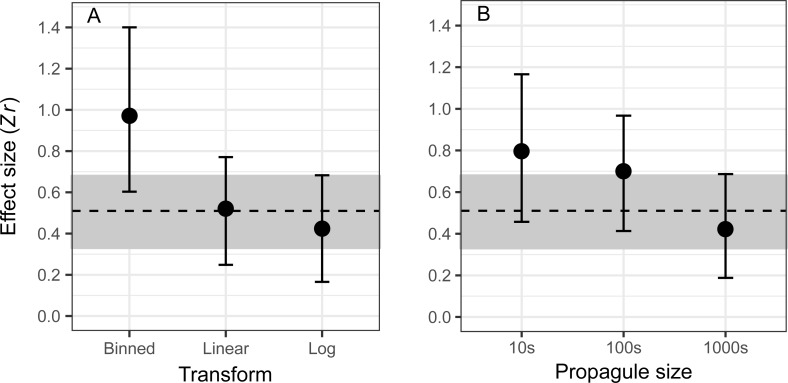
Differences in the effect size for levels of the most influential moderator variables in explaining heterogeneity in the ‘Propagule size’ dataset (*n* = 56; Δ_LOO-IC_ relative to Intercept-only model = 2.81; Table C in S1 Text). Transform (A) is the form of the propagule pressure variable in its measured relationship with establishment success, and Propagule size (B) is the number of individuals in log_10_ increments. Effect size estimates are conditional on the other moderators. The dashed line shows the mean population-level effect size (0.51) for the ‘Propagule size’ dataset, and the grey bands show the 95% CI (0.33–0.68). CI, credible interval; LOO-IC, leave-one-out cross-validation information criterion; *Zr*, Fisher’s z-transformed correlation coefficient.

Focussing our analysis on the subset of studies that examined a direct measure of propagule number or size (‘Metric’ model; i.e., excluding proxy measures; *n* = 70), we found no evidence for any variables explaining heterogeneity among effect sizes (Table B in S1 Text). When we further restricted our analysis to relationships for which information on the number of individuals released is available (‘Propagule size’ model; *n* = 56), there was evidence (Δ_LOO-IC_ > 2.0 from the Intercept-only model) that effect size varied according to how propagule size was treated for analysis (i.e., ‘Transform’; linear, log transformed, or binned) and the range over which ‘Propagule size’ was measured (Fig 2; Table C in S1 Text). For the latter variable, effect size decreased as the range in the number of individuals released increased.

### Variation in relationship shape

**Fig 3:**
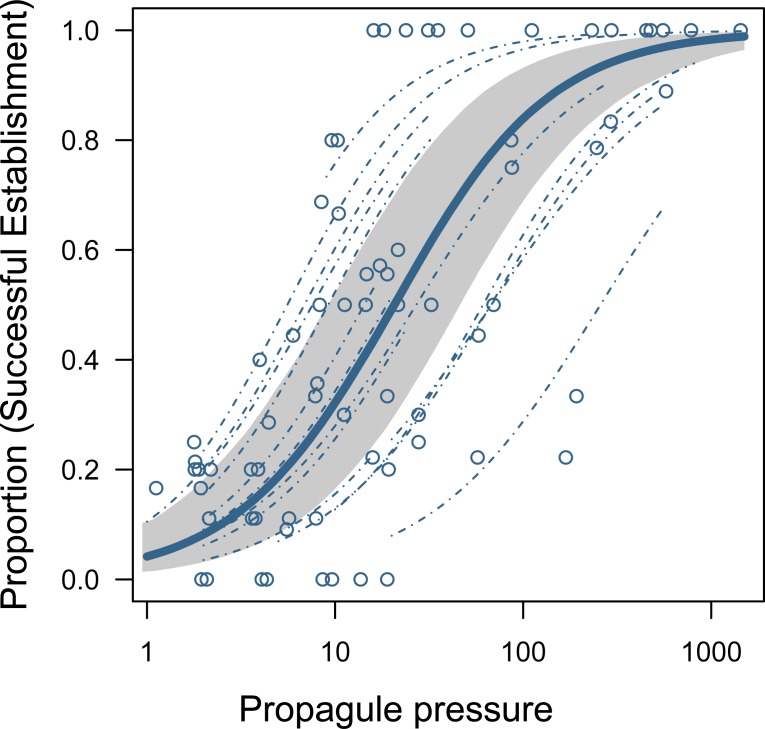
Estimated relationship of establishment success with propagule pressure and 95% CI (shaded). Dashed lines are individual experimental relationships based on a logistic model with random variation in the intercept and slope among individual experiments. Note, there was no statistical evidence for (i) the model with random intercept and slopes performing better than the random Intercept-only model nor for (ii) different slopes between invertebrates and vertebrates (taxon [= *n*]; invertebrate = 9, vertebrate = 5; slope difference = 0.13, 95% CI = -0.9–1.2). Data points are raw data from 14 relationships from 11 studies (see S2 Text) that experimentally tested associations between propagule size and establishment probability (see Materials and methods for more details). CI, credible interval.

For the 11 studies that provided raw data on the binary outcome of establishment success, we found a strong positive relationship between propagule size and the probability of establishment (Fig 3). For this subset of experimental studies, the mean population-level effect size was 2.41 (SD = 0.26; 95% CI = 1.94–2.92). This effect size equates to an 11.1-fold increase in the odds of successful establishment with order of magnitude (log_10_) increases in propagule pressure (95% CI = 7.0–18.5). Establishment success responded most strongly to propagule pressures between 10 and 100 individuals (Fig 3).

## Discussion

In the first quantitative systematic review of the effect of propagule pressure on establishment success, we have presented consistent evidence that alien populations founded by more individuals are more likely to establish. Indeed, we have shown that the mean propagule pressure effect is clearly positive and that this was a feature of every subset of the data we analysed.

The details of the propagule pressure relationship with establishment success are as important to the management of invasions, and to our understanding of small-population dynamics, as is the direction of the relationship. The shape of the relationship informs at what sizes populations are most subject to stochastic and other factors that increase extinction risk [50], and it informs the risk-reduction payoff associated with reducing alien species releases via biosecurity policy [11]. As Fig 3 illustrates, there is remarkable consistency across experimental studies in the rate at which establishment probability increases with incremental increases in the size of the founding population. Success in these experimental studies seems most critically to depend on propagule pressures in the range of 10–100 individuals: establishment probability is low for founding populations at the lower end of this range but highly likely (but never certain) for founding populations at the upper end. Similar values have also been shown to be the critical range for bird introductions worldwide [20]. This result suggests that it does not take many individuals to found an alien population and that management needs to reduce alien species releases to very low levels (essentially into single figures) to have some confidence that establishment is unlikely. Even then, the risk is not eliminated (see e.g., [47])—only complete exclusion of an alien can guarantee no establishment. Fig 3 also illustrates that, even though small numbers of individuals (e.g., <10) can sometimes lead to relatively high probabilities of establishment success (e.g., >0.8), this does not negate the universality of the propagule pressure effect, as has sometimes been suggested [40].

The utility of a quantitative meta-analytical approach is that it can identify heterogeneity in effect sizes, and it can potentially identify sources of that heterogeneity. To the latter end, we tested whether a range of methodological, analytical, and biological differences in the set of studies analysed can explain observed variation. Our findings are as interesting for where heterogeneity does not reside as for where it does. Most notably, none of the analyses produced models with strong support (relative model weights from LOO-IC) compared with an Intercept-only model. Variation was not accounted for, in any analysis, by the spatial scale of study (from local to global), and there was no evidence for an effect of the source of evidence (experimental, observational, or proxy). The effect of propagule pressure on establishment success manifests equally, as positive, within local experimental tests [51] and global compilations [29]. We also found the propagule pressure effect to be just as strong when studies used proxies for propagule pressure rather than direct measurements of the number of individuals released.

Heterogeneity in effect sizes was most apparent when taking account of the variety of ways analyses were performed across studies. The moderator variable that most consistently explained variation in effect size was whether analysis was univariate or multivariate (see Tables A and C in S1 Text). Multivariate analyses produced noticeably weaker effects of propagule pressure in the full dataset (Fig 1). This effect may result because propagule pressure is acting in part as a proxy for variables that explain variation in alien population establishment success (e.g., annual fecundity [32]), so studies that include these variables in a statistical model will reduce (albeit not remove) the effect of propagule pressure. However, there is also a more prosaic reason for the univariate/multivariate difference. Estimates of the variance explained by a predictor variable depend on the amount of total variance to be explained, and including additional predictor variables inevitably reduces unexplained variance. Unless predictor variables are completely uncorrelated in such models, effect size estimates for a given variable, such as propagule pressure, will be reduced, according to which other predictor variables are in the model [52].

We also found that if analysis is restricted only to those studies that could directly measure the number of individuals released (i.e., that analyse propagule size), then the positive relationship between propagule pressure and establishment success is strongest when large step changes across ranges of propagule pressures are discretised, compared with continuous (either linear or log-transformed) measures (Fig 2A; Table 1; Table C in S1 Text). Bins can span a variety of ranges in propagule pressure analyses, and there is an obvious difficulty in comparing effect size estimates for discretised relationships where the cut points differ between studies. The choice can also affect the result obtained. We caution against the practice of discretising continuous variables, particularly where there is not well-trodden a priori categorisation (see also [53]). Furthermore, effect estimates based on cutoffs that are not defined a priori will be biased, and ordinary inferential statistics will be overly optimistic regarding the influence of propagule pressure on establishment [54].

Effect sizes also weakened as the range of release sizes (i.e., propagule size) increases (Fig 2B; Table 1; Table C in S1 Text). Effect sizes were strongest for studies where minimum to maximum release sizes spanned around 100 individuals or fewer. Propagule ranges of this magnitude are likely primarily to concern the lower end of release sizes, which is exactly the range over which increases in propagule size have their strongest impact in experimental studies in our data (Fig 3) and within other analyses (see, e.g., [20]). Small propagules are highly likely to fail to establish, but the average probability of success is already close to its maximum value (always <1) by the time 100 individuals have been released. Our analyses suggest that increasing the range of release sizes beyond approximately 100 individuals may therefore dilute the measured propagule pressure effect, i.e., by including a large number of release sizes that generally do not explain more variation in success [55].

Finally, we explicitly accounted for the nonindependent analysis of overlapping datasets by grouping studies and effect sizes which relied on the same (or similar) data. Although we found some evidence that vertebrate and invertebrate studies have larger effect sizes than plant studies (Table A in S1 Text)—perhaps because the ability of many successful alien plant species to reproduce asexually or vegetatively may mitigate the effects of small population sizes relative to most animal species—there was no clear effect of any of the other groupings. In particular, the Acclimatisation Society introduction of birds to New Zealand (i.e., the ‘Kiwi’ effect) produced a strong effect size (0.74; 95% CI = 0.48–0.99) but one that overlapped the overall mean population effect size. The acclimatisation of alien vertebrates in New Zealand has provided a compelling case study of the invasion process, in part because the island provides one of the best-documented natural experiments in the establishment of alien birds and mammals. However, there is no evidence that the influence of propagule pressure in driving establishment success in New Zealand is notably larger than elsewhere in the wold.

In conclusion, we find consistent support for a positive relationship between propagule pressure and establishment success despite a priori expectations that the effect magnitude (or sign) of this relationship will vary according to a variety of biological and methodological factors. There is heterogeneity in effect sizes, but this is primarily associated with different analytical approaches. Our analyses dispel suggestions that the effect of propagule pressure on establishment success is an artefact of the analyses of only certain data (e.g., historical), conducted in only certain ways (e.g., experimental), on certain metrics of propagule pressure (e.g., pressure, size, or number), or when the analysis focuses only on certain species (e.g., vertebrates) or certain locations (e.g., New Zealand). There was no evidence for publication bias, and the propagule pressure effect is remarkably consistent in both size and shape. There are very few, if any, other factors purported to explain establishment success that can claim such universal support [13]. In a field that seeks to address one of the more pressing issues of global change (invasive species [56]), this result is encouraging as it provides a clear policy and management target for slowing invasion rates: reduce propagule pressure, ideally to single figures or zero, regardless of any other feature of the invasion.

## Materials and methods

### Data

We searched Web of Science, Biosis, and EbscoHost using the following search terms: propagule AND pressure AND ecolog*, ‘introduction effort’ AND (ecolog* OR invas*), propagule AND pressure AND invas*, propagule AND (size OR number) AND (ecolog* OR invas*), pressure AND invas* AND ecolog*, ‘establishment success’ AND propagule. We varied this search string and included all generally used synonyms of ‘alien’ including ‘exotic’, ‘non-native’, ‘non-indigenous’, and ‘naturalized’ [30]. In addition, backwards and forwards searches from citations of relevant papers were conducted from three key highly cited and well-known peer-reviewed publications of propagule pressure [11, 14, 57].

Our initial search identified approximately 3,000 unique papers (see Fig A in S1 Text), most of which (74.3%) were not directly relevant as they, for example, (i) referred specifically to medical or other unrelated literature; (ii) were literature reviews, opinion articles, or pertained to invasion policy; or (iii) invoked propagule pressure while providing no analysis for the effect itself. This left 769 papers that were likely to be of high relevance and were thus closely evaluated. We assessed these papers according to the six criteria listed in the Supporting Information (S1 Text). A further 713 studies were excluded because of one (or more) of these criteria. The majority of these papers (70%) were excluded because they used genetic methods to deduce propagule pressure or because they evaluated the role of propagule pressure in the geographical spread of an alien species and not at initial establishment. This left a total of 56 studies (years 1986–2016 inclusive) reporting statistical analyses of the relationship between propagule pressure and establishment success (see S1 Data; S3 Text). We relied on author assessments of establishment success for the populations they tracked, which were universally reported as a binary outcome (success, failure). Some of these papers provided multiple results, which could be from analyses with or without certain groups of species (or individuals) included (e.g., acclimatisation efforts from different regions of the world [32]), or from different datasets presented in a single paper (e.g., different insect taxa [58]). In total, the 56 studies reported the results for 96 different relationships between propagule pressure and establishment success (see S1 Data). A number of studies repeated analyses on the same or overlapping dataset (the most common being Acclimatisation Society introductions of birds to New Zealand; the ‘Kiwi’ effect, *n* = 8 studies). We grouped these studies, which were based on a common underlying dataset, so that the 96 relationships were analysed across 43 study/dataset groups (see Fig B in S1 Text). For each of these relationships, we scored the following moderator variables:

1. Taxon: We classified taxon as either plant (*n* = 10), invertebrate (*n* = 25), or vertebrate (*n* = 61).
2. Spatial scale: We classified analyses on the basis of the spatial extent over which data were observed; local (*n* = 5), state (*n* = 13), country (*n* = 31), continental (*n* = 20), and global (*n* = 27). State indicates any spatial unit nested within a country (e.g., a British county or US state), and local refers to field and mesocosm studies.
3. Methodology: We distinguished whether the analysis used a ‘direct’ measure of propagule pressure (i.e., propagule size or propagule number; sensu [11]) (*n* = 70) or a ‘proxy’ variable (*n* = 26). For ‘direct’ measures of propagule pressure, we scored the type of scientific methodology as either experimental (*n* = 11) or observational (*n* = 59). Observational studies mainly related to data on the success or failure of historical introductions. Examples of proxies included the number of 19th century plant catalogues selling a species [59] and the species’ abundance in the Taiwanese bird market trade [60].
4. Number of predictors: We scored effect sizes according to whether they were taken from a model that included other explanatory variables, i.e., univariate (*n* = 62) or multivariate models (*n* = 34).
5. Transform: We classified analyses according to how they treated the form of the propagule pressure data in its measured relationship with establishment success, i.e., linear (*n* = 35), log transformed (usually log_10_, but not always; e.g., [29]) (*n* = 40), or binned into a small number of ordinal categories of varying size (*n* = 21).
6. Propagule pressure (*n* = 70): We distinguished whether the analysis used an estimate of propagule size (i.e., individuals; *n* = 56) or propagule number (i.e., events; *n* = 14). Note, this applies only to the ‘direct’ subset of the Proxy variable.
7. Propagule size (*n* = 56): For studies of propagule size, we recorded the range (in the number of individuals) across which establishment success was measured, i.e., tens (*n* = 12), hundreds (*n* = 19), or thousands (*n* = 25).

### Effect size calculation

The majority of studies (41 out of 56) conducted a logistic- or probit-type regression analysis between establishment success and propagule pressure, in which individual success was depicted as a binary outcome (0 = failed introduction, 1 = successful introduction); additional test statistics are detailed in the Supporting Information (S1 Data). We calculated a (statistical) population-level effect size of propagule pressure on establishment success using information from all 96 relationships given by the 43 study/dataset groups. Our effect-size response variable was the Fisher’s z-transformed correlation coefficient, *Zr* [61]. We converted test statistics to correlation coefficients (*r*) following the algebraic recalculations and detailed conversions provided in Box 13.3 by Lajeunesse and colleagues [62].

### Bayesian meta-regression analysis

We conducted a Bayesian multilevel meta-analysis to estimate the overall statistical population-level effect size. This approach allowed us to estimate the heterogeneity in effect size for the random effects: among studies and among relationships within a study [63]. Heterogeneity at each level of the random effects was measured using I^2^ statistics [64, 65]. We then fitted multilevel meta-regression models with these same structured random effects, which included moderator variables to examine their contribution to explaining the observed heterogeneity in effect sizes. These candidate models fitted to the full dataset included the following moderators (singly, or at most in pairs, as explained below): (i) ‘Taxon’, (ii) ‘Spatial scale’, (iii) ‘Methodology’, (iv) ‘Number of predictors’, and (v) ‘Transform’.

### Additional moderators

There were two additional moderator variables that we had reason to believe would account for heterogeneity in population-level effect size—moderator variables ‘Propagule pressure’ and ‘Propagule size’. In order to evaluate the influence of these variables, we subset the reported relationships into groups, with each subset containing only relationships that had information on the focal moderator variable of interest (i.e., excluding ‘Proxy’ studies). The first subgroup considered whether the component of propagule pressure (i.e., propagule size or propagule number) explained heterogeneity in effect sizes (two-level categorical variable; *n* = 70). The second evaluated the extent to which effect size was influenced by the range across which propagule size was measured (tens, hundreds, or thousands of individuals; *n* = 56).

### Model selection and cross-validation

For all models, regardless of subset or analytical underpinning, we included either single moderator variables or the additive effects of pairs of moderators, provided that there was sufficient replication (*n* ≥ 3) at all cross-classification levels of the pairs of moderator variables. We imposed these constraints to avoid overfitting due to limited overall number of effect size measures available. We used a LOO-IC to estimate the predictive accuracy of the Bayesian hierarchical meta-regression models [66]. We calculated relative model weights based on the LOO-IC for each set of candidate models. We adopted the same rules of thumb commonly used for Akaike's information criterion model weights [67] to evaluate model rankings (Δ_LOO-IC_ > 2.0).

### Statistical analysis and publication bias

Statistical analyses were conducted using the R (v.3.1.0) software environment for statistical and graphical computing [68]. We used forest plots [69] to display estimated effect sizes associated with individual studies, and error bar plots were used to display marginal means for levels of the categorical moderator variables. Posterior predictions for each moderator were calculated with the other moderators constrained to their reference levels and with measurement error fixed at the median observed value. All Bayesian models were fitted using the open-source software Stan [70, 71] and the R package brms [72]. Sampling was conducted for 20,000 iterations of each of 3 chains with a burn-in of 1,000 samples. Moderator effects used an improper flat prior. The random effect variance components used a half Student *t* test prior.

Selective reporting of results can lead to ‘publication bias’ [73]. We investigated for evidence of publication bias (visually and statistically) by examining the degree of asymmetry in the residual effect sizes from the highest-ranked model fitted to the full dataset (Table 1). This model includes the moderator variable 'number of predictors' and random effects among author/dataset and within-study variation, plus the measurement error (see Results), as the data points in the funnel plot.

Egger’s regression provides an inferential test to examine evidence against the hypothesis of no publication bias, but this test requires independent observed effect sizes. Nakagawa and Santos [63] present a modified Egger’s regression based on these meta-analytic residuals weighted by their inverse precision, and we use this approach statistically to examine publication bias.

### Variation in relationship shape

Some studies have used an experimental approach to evaluate how incrementally increasing propagule size served to alter establishment probability. Each experiment consisted of a series of populations held in either constant or systematically altered environments. The treatment applied is the varying of founding numbers of individuals across replicate populations, with the range of founder sizes determined by the researchers. Establishment probability in this context is the number of experimental populations that remained extant (successful) after a predefined time frame (>1 generation) out of the total number of experimental populations initially constructed. Eleven of these studies provided sufficient raw data for us to extract and combine 14 relationships into a single analysis that evaluates the specific variation in the shape of the propagule pressure effect.

We used a Bayesian generalised linear mixed model with a logit link and binomial variance function to evaluate the slope of the effect of propagule pressure on establishment probability, accounting for average differences in establishment success between experiments (a random effect). We also examined evidence for random differences in the slope of the relationship across experiments. Propagule pressure was log_10_ transformed and centred for this analysis. We found no evidence for overdispersion in the binomial proportions after accounting for the random effects.

## Supporting information

S1 Text Supplementary methods and results. (DOCX)

S2 Text References for the experimental analysis in Fig 3. (DOCX)

S3 Text References for S1 Data. (DOCX)

S1 Data Full data file for analysis. (CSV)

## Abbreviations

CI: credible interval
LOO-IC: leave-one-out cross-validation information criterion
Zr: Fisher’s z-transformed correlation coefficient
