# Supplementary material for: Dissecting the null model for biological invasions: A meta-analysis of the propagule pressure effect
Source: PLoS Biol. 2018 Apr 23;16(4):e2005987. doi: 10.1371/journal.pbio.2005987 (PMC5933808; doi:10.1371/journal.pbio.2005987)
Supplement: S1 Text — (DOCX) [file pbio.2005987.s001.docx]

**S1 Text: *Supplementary Methods and Results***

Of the 769 papers that were identified as likely to be of high relevance, and were assessed for eligibility, 713 were excluded because of one of the following criteria (see Fig A):

1. *Genetic* *(n = 166)*: These studies produced estimates of initial population size (propagule size) using inferential statistics based on standing genetic variation in the current population (e.g., Dlugosch & Parker 2008; Arntzen *et al.* 2010; Consuegra *et al.* 2011). In the majority of such analysis only one population was assessed, thus providing no information on how relative differences in propagule pressure influenced establishment success. When propagule size was assessed for more than one population there was no attempt to generate statistical relationships (effect sizes) between size and establishment.
2. *Germination/Recruitment (n = 53)*: The authors examined germination or recruitment rates, or the survival, of newly planted, seeded or settled individuals within a single generation (e.g., Eckberg *et al.* 2012; Eckstein *et al.* 2012). While these studies inform about components of establishment they do not provide an accurate estimate of whether the new population is self-sustaining. We suggest that this collection of research, which is substantial and growing, deserves to be the subject of its own meta-analysis.
3. *Lab/Native (n = 13)*: The study did not introduce individuals of an alien species (e.g., Vercken *et al.* 2013).
4. *No Data or No Effect Size (n = 43)*: The authors neither performed statistical analysis on the relationship between propagule pressure data and establishment success, nor presented raw data to enable such analysis. This also includes studies for which insufficient information was provided to recalculate an effect size (see *Effect size calculation* in Main Text).
5. *Not Establishment* *(n = 371)*: The authors did not address the establishment phase of the invasion pathway as described by Blackburn *et al.* (2011). For example, a number of papers considered other stages of the invasion pathway, such as escape from captivity (e.g., Tingley *et al.* 2011) or the spread of already established populations into adjacent areas (e.g., Pyšek *et al.* 2008). Although relevant in other contexts, they did not address our *a priori* question of the role of propagule pressure in determining initial establishment success of alien populations.
6. *Simulation/Fitting* *(n = 67)*: The authors presented only simulation models of establishment, or fitted distributions to existing data without any statistical analysis (e.g., Duncan *et al.* 2014).

These exclusions left a total of 56 studies, reporting the results for 96 different relationships between propagule pressure and establishment success.

**
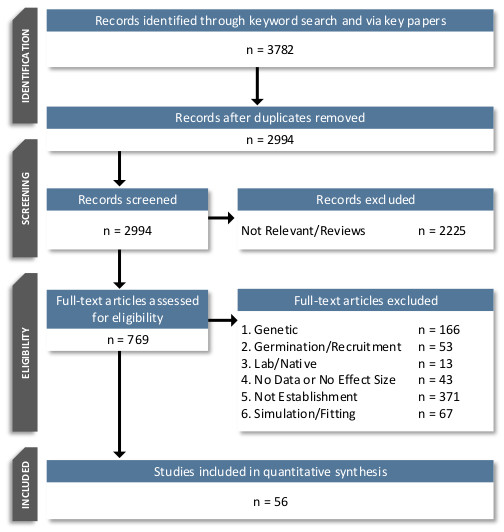
**

**Fig A.** PRISMA flow chart (*sensu* Moher *et al.* 2010) for the inclusion (or exclusion) of primary studies in the quantitative meta-analysis of the influence of propagule pressure on invasion establishment success. Records were initially identified from Web of Science, Biosis, and EbscoHost databases. Additional records were identified from backwards and forwards searches, in Web of Science, of key References: Williamson (1996); Lockwood *et al.* (2005); Simberloff (2009). Records were first excluded based on a mismatch in the Abstract or scientific discipline. Full-text articles were excluded based on the detailed (six) reasons provided in the Methods, and summarised here under *Eligibility* (Full-text articles excluded; 1-6).

**
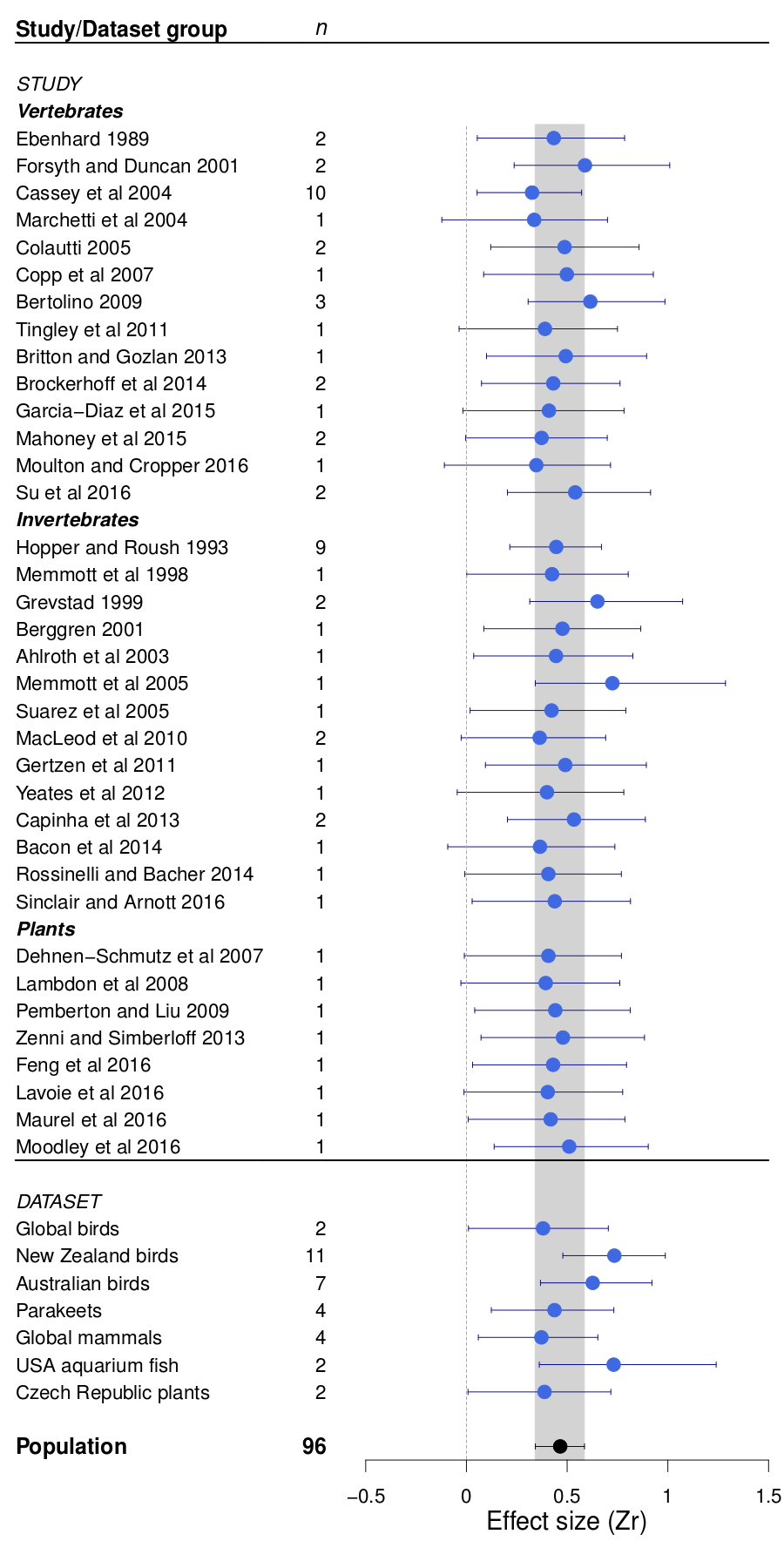
**

**Fig B.** Heterogeneity in effect size among studies. The mean population-level effect size (0.47) is displayed at the bottom of the Figure, and grey bands show the 95% credible interval (0.34, 0.59). The dotted vertical line denotes *Zr* = 0. Note, study authors (bibliographic citations) dataset groups (see Main Text article), and all accompanying information, are provided in S3 Text and S1 Data, respectively.

**
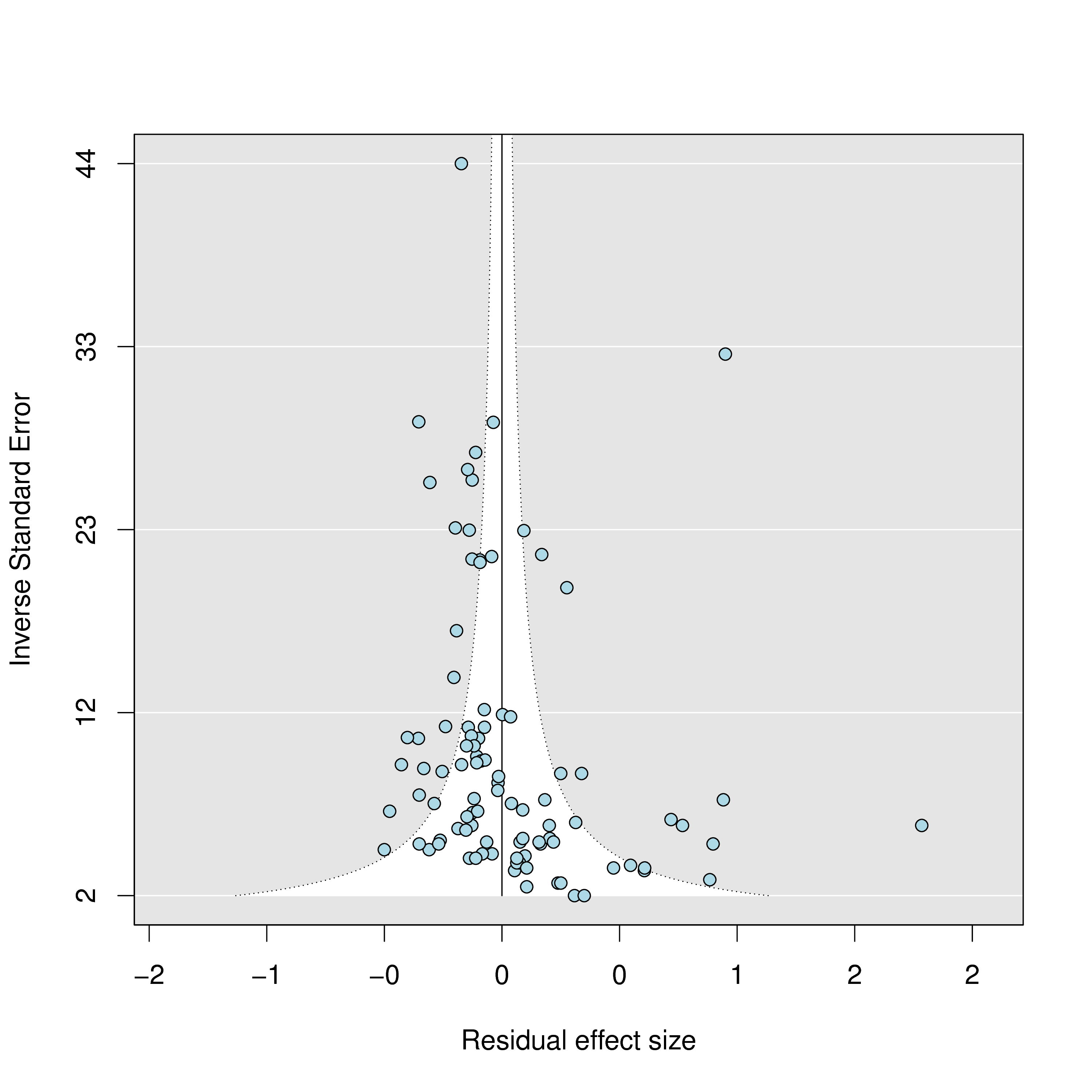
**

**Fig C.** Funnel plot of residual effect sizes, from the highest ranked model fitted to the full dataset (Table 1), plotted against their inverse precision (Standard Error).

**Table A.** Model selection summary statistics based on leave-one-out cross-validation information criterion (LOO-IC) for the overall effect size model (n = 96). Definitions for the moderator labels follow those used in the main text.

| Model moderators | LOO-IC | Relative weights |
| --- | --- | --- |
| *Number of predictors* | 22.14 | 0.425 |
| *Taxon + Number of predictors* | 23.72 | 0.193 |
| *Methodology* + *Number of predictors* | 25.18 | 0.093 |
| Intercept-only | 25.54 | 0.077 |
| *Taxon* | 25.55 | 0.077 |
| *Transform* | 25.86 | 0.066 |
| *Transform + Number of predictors* | 27.20 | 0.034 |
| *Methodology* | 28.41 | 0.018 |
| *Spatial scale* | 28.65 | 0.016 |

**Table B.** Model selection summary statistics based on leave-one-out cross-validation information criterion (LOO-IC) for the ‘*Metric*’ (i.e., propagule size or propagule number) model subset (n = 70). Definitions for the moderator labels follow those used in the main text.

| Model moderators | LOO-IC | Relative weights |
| --- | --- | --- |
| Intercept-only | 35.44 | 0.147 |
| *Spatial scale* | 35.82 | 0.121 |
| *Transform + Number of predictors* | 35.87 | 0.118 |
| *Transform* | 35.89 | 0.117 |
| *Propagule pressure + Number of predictors* | 36.27 | 0.097 |
| *Propagule pressure + Transform* | 36.30 | 0.095 |
| *Propagule pressure* | 36.50 | 0.086 |
| *Number of predictors* | 36.91 | 0.070 |
| *Taxon* | 37.33 | 0.057 |
| *Methodology + Number of predictors* | 37.53 | 0.052 |
| *Methodology* | 38.02 | 0.040 |

**Table C.** Model selection summary statistics based on leave-one-out cross-validation information criterion (LOO-IC) for the ‘*Propagule size’* model subset (n = 56). Definitions for the moderator labels follow those used in the main text.

| Model moderators | LOO-IC | Relative weights |
| --- | --- | --- |
| *Transform + Propagule size* | 31.88 | 0.254 |
| *Transform* + *Number of predictors* | 32.35 | 0.201 |
| *Transform* | 33.57 | 0.109 |
| *Propagule size* | 34.22 | 0.079 |
| *Number of predictors* | 34.35 | 0.074 |
| *Methodology* | 34.39 | 0.072 |
| *Methodology + Number of predictors* | 34.66 | 0.063 |
| Intercept-only | 34.69 | 0.062 |
| *Spatial scale* | 35.30 | 0.046 |
| *Taxon* | 35.60 | 0.039 |

**References**

1. Arntzen, J.W., Burke, T. & Jehle, R. (2010). Estimating the propagule size of a cryptogenic crested newt population. *Anim Conserv*, 13, 74-81.
2. Blackburn, T.M., Pyšek, P., Bacher, S., Carlton, J.T., Duncan, R.P., Jarošík, V. *et al.* (2011). A proposed unified framework for biological invasions. *Trends Ecol Evol*, 26, 333-339.
3. Consuegra, S., Phillips, N., Gajardo, G. & de Leaniz, C.G. (2011). Winning the invasion roulette: escapes from fish farms increase admixture and facilitate establishment of non-native rainbow trout. *Evol Appl*, 4, 660-671.
4. Dlugosch, K.M. & Parker, I.M. (2008). Founding events in species invasions: genetic variation, adaptive evolution, and the role of multiple introductions. *Mol Ecol*, 17, 431-449.
5. Duncan, R.P., Blackburn, T.M., Rossinelli, S. & Bacher, S. (2014). Quantifying invasion risk: the relationship between establishment probability and founding population size. *Methods Ecol Evol*, 5, 1255-1263.
6. Eckberg, J.O., Tenhumberg, B. & Louda, S.M. (2012). Insect herbivory and propagule pressure influence *Cirsium vulgare* invasiveness across the landscape. *Ecology*, 93, 1787-1794.
7. Eckstein, R.L., Ruch, D., Otte, A. & Donath, T.W. (2012). Invasibility of a nutrient-poor pasture through resident and non-resident herbs is controlled by litter, gap size and propagule pressure. *PloS ONE*, 7, e41887.
8. Lockwood, J.L., Cassey, P. & Blackburn, T. (2005). The role of propagule pressure in explaining species invasions. *Trends Ecol Evol*, 20, 223-228.
9. Moher, D., Liberati, A., Tetzlaff, J., Altman, D.G. & Group, P. (2010). Preferred reporting items for systematic reviews and meta-analyses: the PRISMA statement. *Int J Surg*, 8, 336-341.
10. Pyšek, P., Jarošík, V., Müllerová, J., Pergl, J. & Wild, J. (2008). Comparing the rate of invasion by *Heracleum mantegazzianum* at continental, regional, and local scales. *Divers Distrib*, 14, 355-363.
11. Simberloff, D. (2009). The role of propagule pressure in biological invasions. *Annu Rev Ecol Evol Syst*, 40, 81-102.
12. Tingley, R., Phillips, B.L. & Shine, R. (2011). Establishment success of introduced amphibians increases in the presence of congeneric species. *Am Nat,* 177, 382-388.
13. Vercken, E., Vincent, F., Mailleret, L., Ris, N., Tabone, E. & Fauvergue, X. (2013). Time‐lag in extinction dynamics in experimental populations: evidence for a genetic Allee effect? *J Anim Ecol*, 82, 621-631.
14. Williamson, M. (1996). *Biological invasions*. Chapman and Hall, London, 244.
