# Supplementary material for: Dissecting the null model for biological invasions: A meta-analysis of the propagule pressure effect
Source: PLoS Biol. 2018 Apr 23;16(4):e2005987. doi: 10.1371/journal.pbio.2005987 (PMC5933808; doi:10.1371/journal.pbio.2005987)
Supplement: S2 Text — (DOCX) [file pbio.2005987.s002.docx]

**S2 Text: *References for the experimental analysis in Fig 3.***

1. Ahlroth, P., Alatalo, R.V., Holopainen, A., Kumpulainen, T. & Suhonen, J. (2003). Founder population size and number of source populations enhance colonization success in waterstriders. *Oecologia*, 137, 617-620, doi: 10.1007/s00442-003-1344-y.
2. Berggren, A. (2001). Colonization success in Roesel's bush-cricket *Metrioptera roeseli*: The effects of propagule size. *Ecology*, 82, 274-280, doi: 10.1890/0012-9658(2001)082[0274:csirsb]2.0.co;2.
3. Britton, J.R. & Gozlan, R.E. (2013). How many founders for a biological invasion? Predicting introduction outcomes from propagule pressure. *Ecology*, 94, 2558-2566, doi: 10.1890/13-0527.1.
4. Ebenhard, T. (1989). Bank vole [*Clethrionomys glareolus* (Schreber, 1780)] propagules of different sizes and island colonization. *J Biogeogr*, 16, 173-180, doi: 10.2307/2845091.
5. Fauvergue, X., Malausa, J.-C., Giuge, L. & Courchamp, F. (2007). Invading parasitoids suffer no Allee effect: A manipulative field experiment. *Ecology*, 88, 2392-2403, doi: 10.1890/06-1238.1.
6. Gertzen, E.L., Leung, B. & Yan, N.D. (2011). Propagule pressure, Allee effects and the probability of establishment of an invasive species (*Bythotrephes longimanus*). *Ecosphere*, 2, 1-17, doi: 10.1890/es10-00170.1.
7. Grevstad, F.S. (1999). Experimental invasions using biological control introductions: the influence of release size on the chance of population establishment. *Biol Invasions*, 1, 313-323, doi: 10.1023/a:1010037912369.
8. Memmott, J., Craze, P.G., Harman, H.M., Syrett, P. & Fowler, S.V. (2005). The effect of propagule size on the invasion of an alien insect. *J Anim Ecol*, 74, 50-62, doi: 10.1111/j.1365-2656.2004.00896.x.
9. Memmott, J., Fowler, S.V. & Hill, R.L. (1998). The EVect of release size on the probability of establishment of biological control agents: Gorse thrips (*Sericothrips staphylinus*) released against gorse (*Ulex europaeus*) in New Zealand. *Biocontrol Sci Techn*, 8, 103-115, doi: 10.1080/09583159830478.
10. Newsome, A.E. & Noble, I.R. (1986). Ecological and physiological characters of invading species. In: *Ecology of Biological Invasions* (eds. Groves, R & Burdon, J). Cambridge University Press, pp. 1-20.
11. Sinclair, J.S. & Arnott, S.E. (2016). Strength in size not numbers: propagule size more important than number in sexually reproducing populations. *Biol Invasions*, 18, 497-505, doi: 10.1007/s10530-015-1022-0.
