# Supplementary material for: Dissecting the null model for biological invasions: A meta-analysis of the propagule pressure effect
Source: PLoS Biol. 2018 Apr 23;16(4):e2005987. doi: 10.1371/journal.pbio.2005987 (PMC5933808; doi:10.1371/journal.pbio.2005987)
Supplement: S3 Text — (DOCX) [file pbio.2005987.s003.docx]

**S3 Text: *References for S1 Data.***

1. Ahlroth, P., Alatalo, R.V., Holopainen, A., Kumpulainen, T. & Suhonen, J. (2003). Founder population size and number of source populations enhance colonization success in waterstriders. *Oecologia*, 137, 617-620, doi: 10.1007/s00442-003-1344-y.
2. Bacon, S.J., Aebi, A., Calanca, P. & Bacher, S. (2014). Quarantine arthropod invasions in Europe: the role of climate, hosts and propagule pressure. *Divers Distrib*, 20, 84-94, doi: 10.1111/ddi.12149.
3. Berggren, A. (2001). Colonization success in Roesel's bush-cricket *Metrioptera roeseli*: The effects of propagule size. *Ecology*, 82, 274-280, doi: 10.1890/0012-9658(2001)082[0274:csirsb]2.0.co;2.
4. Bertolino, S. (2009). Animal trade and non-indigenous species introduction: the world-wide spread of squirrels. *Divers Distrib*, 15, 701-708, doi: 10.1111/j.1472-4642.2009.00574.x.
5. Blackburn, T.M., Prowse, T.A.A., Lockwood, J.L. & Cassey, P. (2011). Passerine introductions to New Zealand support a positive effect of propagule pressure on establishment success. *Biodivers Conserv*, 20, 2189-2199, doi: 10.1007/s10531-011-0081-5.
6. Bradie, J., Chivers, C. & Leung, B. (2013). Importing risk: quantifying the propagule pressure-establishment relationship at the pathway level. *Divers Distrib*, 19, 1020-1030, doi: 10.1111/ddi.12081.
7. Britton, J.R. & Gozlan, R.E. (2013). How many founders for a biological invasion? Predicting introduction outcomes from propagule pressure. *Ecology*, 94, 2558-2566, doi: 10.1890/13-0527.1.
8. Brockerhoff, E.G., Kimberley, M., Liebhold, A.M., Haack, R.A. & Cavey, J.F. (2014). Predicting how altering propagule pressure changes establishment rates of biological invaders across species pools. *Ecology*, 95, 594-601, doi: 10.1890/13-0465.1.
9. Capellini, I., Baker, J., Allen, W.L., Street, S.E. & Venditti, C. (2015). The role of life history traits in mammalian invasion success. *Ecol Lett*, 18, 1099-1107, doi: 10.1111/ele.12493.
10. Capinha, C., Brotons, L. & Anastácio, P. (2013). Geographical variability in propagule pressure and climatic suitability explain the European distribution of two highly invasive crayfish. *J Biogeogr*, 40, 548-558, doi: 10.1111/jbi.12025.
11. Cardador, L., Carrete, M., Gallardo, B. & Tella, J.L. (2016). Combining trade data and niche modelling improves predictions of the origin and distribution of non-native European populations of a globally invasive species. *J Biogeogr*, 43, 967-978, doi: 10.1111/jbi.12694.
12. Cassey, P., Blackburn, T.M., Sol, S., Duncan, R.P. & Lockwood, J.L. (2004). Global patterns of introduction effort and establishment success in birds. *P Roy Soc Lond B Bio*, 271, S405-S408, doi: 10.1098/rsbl.2004.0199.
13. Colautti, R.I. (2005). Are characteristics of introduced salmonid fishes biased by propagule pressure? *Can J Fish Aquat Sci*, 62, 950-959, doi: 10.1139/f05-002.
14. Copp, G., Templeton, M. & Gozlan, R. (2007). Propagule pressure and the invasion risks of non‐native freshwater fishes: a case study in England. *J Fish Biol*, 71, 148-159, doi: 10.1111/j.1095-8649.2007.01680.x.
15. Dehnen-Schmutz, K., Touza, J., Perrings, C. & Williamson, M. (2007). The horticultural trade and ornamental plant invasions in Britain. *Conserv Biol*, 21, 224-231, doi: 10.1111/j.1523-1739.2006.00538.x.
16. Duggan, I.C., Rixon, C.A.M. & MacIsaac, H.J. (2006). Popularity and propagule pressure: determinants of introduction and establishment of aquarium fish. *Biol Invasions*, 8, 377-382, doi: 10.1007/s10530-004-2310-2.
17. Duncan, R.P. (1997). The role of competition and introduction effort in the success of passeriform birds introduced to New Zealand. *Am Nat*, 149, 903-915, doi: 10.1086/286029.
18. Duncan, R.P., Bomford, M., Forsyth, D.M. & Conibear, L. (2001). High predictability in introduction outcomes and the geographical range size of introduced Australian birds: a role for climate. *J Anim Ecol*, 70, 621-632, doi: 10.1046/j.1365-2656.2001.00517.x.
19. Ebenhard, T. (1989). Bank vole [*Clethrionomys glareolus* (Schreber, 1780)] propagules of different sizes and island colonization. *J Biogeogr*, 16, 173-180, doi: 10.2307/2845091.
20. Fautley, R., Coulson, T. & Savolainen, V. (2012). A comparative analysis of the factors promoting deer invasion. *Biol Invasions*, 14, 2271-2281, doi: 10.1007/s10530-012-0228-7.
21. Feng, Y.H., Maurel, N., Wang, Z.H., Ning, L., Yu, F.H. & van Kleunen, M. (2016). Introduction history, climatic suitability, native range size, species traits and their interactions explain establishment of Chinese woody species in Europe. *Global Ecol Biogeogr*, 25, 1356-1366, doi: 10.1111/geb.12497.
22. Forsyth, D.M. & Duncan, R.P. (2001). Propagule size and the relative success of exotic ungulate and bird introductions to New Zealand. *Am Nat*, 157, 583-595, doi: 10.1086/320626.
23. Garcia-Diaz, P., Ross, J.V., Ayres, C. & Cassey, P. (2015). Understanding the biological invasion risk posed by the global wildlife trade: propagule pressure drives the introduction and establishment of Nearctic turtles. *Glob Change Biol*, 21, 1078-1091, doi: 10.1111/gcb.12790.
24. Gertzen, E.L., Leung, B. & Yan, N.D. (2011). Propagule pressure, Allee effects and the probability of establishment of an invasive species (*Bythotrephes longimanus*). *Ecosphere*, 2, 1-17, doi: 10.1890/es10-00170.1.
25. Gonzalez-Suarez, M., Bacher, S. & Jeschke, J.M. (2015). Intraspecific trait variation is correlated with establishment success of alien mammals. *Am Nat*, 185, 737-746, doi: 10.1086/681105.
26. Green, R.E. (1997). The influence of numbers released on the outcome of attempts to introduce exotic bird species to New Zealand. *J Anim Ecol*, 66, 25-35, doi: 10.2307/5961.
27. Grevstad, F.S. (1999). Experimental invasions using biological control introductions: the influence of release size on the chance of population establishment. *Biol Invasions*, 1, 313-323, doi: 10.1023/a:1010037912369.
28. Hopper, K.R. & Roush, R.T. (1993). Mate finding, dispersal, number released, and the success of biological control introductions. *Ecol Entomol*, 18, 321-331, doi: 10.1111/j.1365-2311.1993.tb01108.x.
29. Krivanek, M., Pysek, P. & Jarosik, V. (2006). Planting history and propagule pressure as predictors of invasion by woody species in a temperate region. *Conserv Biol*, 20, 1487-1498, doi: 10.1111/j.1523-1739.2006.00477.
30. Lambdon, P.W., Lloret, F. & Hulme, P.E. (2008). How do introduction characteristics influence the invasion success of Mediterranean alien plants? *Perspect Plant Ecol*, 10, 143-159, doi: 10.1016/j.ppees.2007.12.004.
31. Lavoie, C., Joly, S., Bergeron, A., Guay, G. & Groeneveld, E. (2016). Explaining naturalization and invasiveness: new insights from historical ornamental plant catalogs. *Ecol Evol*, 6, 7188-7198, doi: 10.1002/ece3.2471.
32. MacLeod, C.J., Paterson, A.M., Tompkins, D.M. & Duncan, R.P. (2010). Parasites lost - do invaders miss the boat or drown on arrival? *Ecol Lett*, 13, 516-527, doi: 10.1111/j.1461-0248.2010.01446.x.
33. Mahoney, P.J., Beard, K.H., Durso, A.M., Tallian, A.G., Long, A.L., Kindermann, R.J. *et al.* (2015). Introduction effort, climate matching and species traits as predictors of global establishment success in non-native reptiles. *Divers Distrib*, 21, 64-74, doi: 10.1111/ddi.12240.
34. Marchetti, M.P., Moyle, P.B. & Levine, R. (2004). Invasive species profiling? Exploring the characteristics of non-native fishes across invasion stages in California. *Freshwater Biology*, 49, 646-661, doi: 10.1111/j.1365-2427.2004.01202.x.
35. Maurel, N., Hanspach, J., Kuhn, I., Pysek, P. & van Kleunen, M. (2016). Introduction bias affects relationships between the characteristics of ornamental alien plants and their naturalization success. *Global Ecol Biogeogr*, 25, 1500-1509, doi: 10.1111/geb.12520.
36. Memmott, J., Craze, P.G., Harman, H.M., Syrett, P. & Fowler, S.V. (2005). The effect of propagule size on the invasion of an alien insect. *J Anim Ecol*, 74, 50-62, doi: 10.1111/j.1365-2656.2004.00896.x.
37. Memmott, J., Fowler, S.V. & Hill, R.L. (1998). The EVect of release size on the probability of establishment of biological control agents: Gorse thrips (*Sericothrips staphylinus*) released against gorse (*Ulex europaeus*) in New Zealand. *Biocontrol Sci Techn*, 8, 103-115, doi: 10.1080/09583159830478.
38. Moodley, D., Proches, S. & Wilson, J.R.U. (2016). A global assessment of a large monocot family highlights the need for group-specific analyses of invasiveness. *AoB PLANTS*, 8, plw009, doi: 10.1093/aobpla/plw009.
39. Moulton, M.P. & Cropper, W.P. (2016). Propagule size and patterns of success in early introductions of Chukar Partridges (*Alectoris chukar*) to Nevada. *Evol Ecol Res*, 17, 713-720.
40. Moulton, M.P., Cropper, W.P. & Avery, M.L. (2013). Is propagule size the critical factor in predicting introduction outcomes in passeriform birds? *Biol Invasions*, 15, 1449-1458, doi: 10.1007/s10530-012-0383-x.
41. Moulton, M.P., Cropper, W.P., Moulton, L.E., Avery, M.L. & Peacock, D. (2012). A reassessment of historical records of avian introductions to Australia: no case for propagule pressure. *Biodivers Conserv*, 21, 155-174, doi: 10.1007/s10531-011-0173-2.
42. Newsome, A.E. & Noble, I.R. (1986). Ecological and physiological characters of invading species. In: *Ecology of Biological Invasions* (eds. Groves, R & Burdon, J). Cambridge University Press, pp. 1-20.
43. Pemberton, R.W. & Liu, H. (2009). Marketing time predicts naturalization of horticultural plants. *Ecology*, 90, 69-80, doi: 10.1890/07-1516.1.
44. Pysek, P., Krivanek, M. & Jarosik, V. (2009). Planting intensity, residence time, and species traits determine invasion success of alien woody species. *Ecology*, 90, 2734-2744, doi: 10.1890/08-0857.1.
45. Rossinelli, S. & Bacher, S. (2014). Higher establishment success in specialized parasitoids: support for the existence of trade-offs in the evolution of specialization. *Funct Ecol*, 29, 277-284, doi: 10.1111/1365-2435.12323.
46. Sinclair, J.S. & Arnott, S.E. (2016). Strength in size not numbers: propagule size more important than number in sexually reproducing populations. *Biol Invasions*, 18, 497-505, doi: 10.1007/s10530-015-1022-0.
47. Sol, D., Bacher, S., Reader, S.M. & Lefebvre, L. (2008). Brain size predicts the success of mammal species introduced into novel environments. *Am Nat*, 172, S63-S71, doi: 10.1086/588304.
48. Sol, D. & Lefebvre, L. (2000). Behavioural flexibility predicts invasion success in birds introduced to New Zealand. *Oikos*, 90, 599-605, doi: 10.1034/j.1600-0706.2000.900317.x.
49. Strubbe, D. & Matthysen, E. (2009). Establishment success of invasive ring-necked and monk parakeets in Europe. *J Biogeogr*, 36, 2264-2278, doi: 10.1111/j.1365-2699.2009.02177.x.
50. Su, S., Cassey, P. & Blackburn, T.M. (2016). The wildlife pet trade as a driver of introduction and establishment in alien birds in Taiwan. *Biol Invasions*, 18, 215-229, doi: 10.1007/s10530-015-1003-3.
51. Suarez, A.V., Holway, D.A. & Ward, P.S. (2005). The role of opportunity in the unintentional introduction of nonnative ants. *P Natl Acad Sci USA*, 102, 17032-17035, doi: 10.1073/pnas.0506119102.
52. Tingley, R., Phillips, B.L. & Shine, R. (2011). Establishment success of introduced amphibians increases in the presence of congeneric species. *Am Nat*, 177, 382-388, doi: 10.1086/658342.
53. Vall-llosera, M. & Sol, D. (2009). A global risk assessment for the success of bird introductions. *J Appl Ecol*, 46, 787-795, doi: 10.1111/j.1365-2664.2009.01674.x.
54. Veltman, C.J., Nee, S. & Crawley, M.J. (1996). Correlates of introduction success in exotic New Zealand birds. *Am Nat*, 147, 542-557, doi: 10.1086/285865.
55. Yeates, A.G., Schooler, S.S., Garono, R.J. & Buckley, Y.M. (2012). Biological control as an invasion process: disturbance and propagule pressure affect the invasion success of *Lythrum salicaria* biological control agents. *Biol Invasions*, 14, 255-271, doi: 10.1007/s10530-011-0060-5.
56. Zenni, R.D. & Simberloff, D. (2013). Number of source populations as a potential driver of pine invasions in Brazil. *Biol Invasions*, 15, 1623-1639, doi: 10.1007/s10530-012-0397-4.
